# Supplementary material for: A Systematic Review of Anatomical Variations of the Inferior Thyroid Artery: Clinical and Surgical Considerations
Source: Diagnostics (Basel). 2025 Jul 23;15(15):1858. doi: 10.3390/diagnostics15151858 (PMC12345916; doi:10.3390/diagnostics15151858)
Supplement: Supplementary file 1 [file diagnostics-15-01858-s001.zip › diagnostics-3626176-supplementary.pdf]

Supplementary Materials

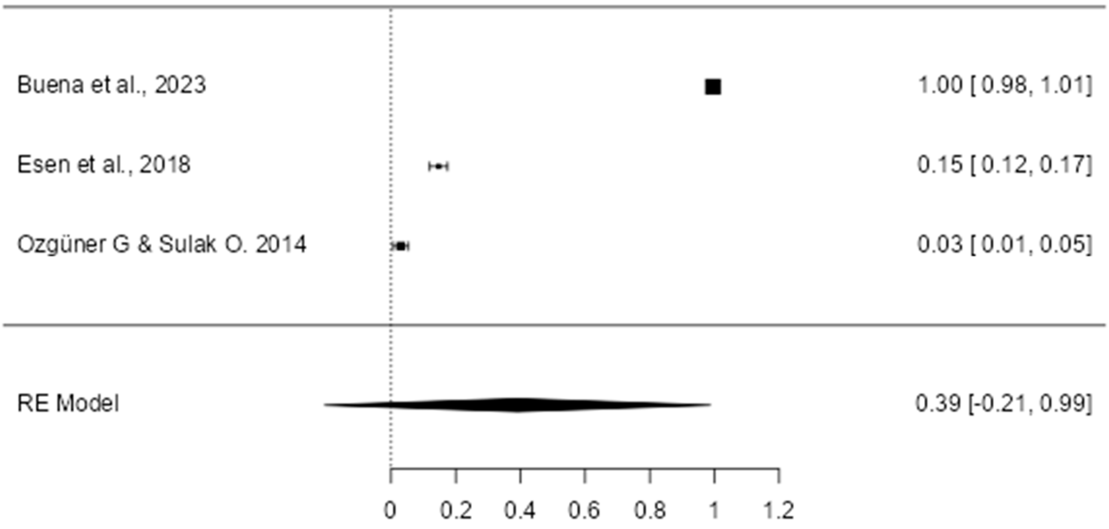

Figure S1. Forest plot of the ITA prevalence in the included articles.

Table S1. Searches strategies.

Database	Search strategy	Results
MEDLINE	"anatomical variations"[Title/Abstract] AND "inferior thyroid artery"[Title/Abstract]	23
MEDLINE		
MEDLINE		
SCOPUS	inferior AND thyroid AND artery AND variations	193
LILACS		
CINHAL	inferior thyroid artery AND variants	5
CINHAL	inferior thyroid artery AND variations	19
	Total	1,778

* All searches were carried out on January 20th, 2024.
